# Evaluation of radiographers’ knowledge and attitudes of image quality optimisation in paediatric digital radiography in Saudi Arabia and Australia: a survey‐based study

**DOI:** 10.1002/jmrs.366

**Published:** 2019-11-06

**Authors:** Haney Alsleem, Rob Davidson, Bandar Al‐Dhafiri, Raghad Alsleem, Hussain Ameer

**Affiliations:** ^1^ Imam Abdulrahman Bin Faisal University Dammam Saudi Arabia; ^2^ University of Canberra Canberra Australia; ^3^ King Faisal University Al‐Hasa Saudi Arabia

**Keywords:** Medical imaging, peadiatric radiography, radiographers' knowledge, quaility assessment

## Abstract

**Introduction:**

Digital radiography (DR) systems enable radiographers to reduce the radiation dose to patients while maintaining optimised image quality. However, concerns still exist about paediatric patients who may be exposed to an increased level of radiation dose which is not needed for clinical practice. The purpose of this study was to evaluate the knowledge, awareness and attitudes, in terms of image quality optimisation of radiographers undertaking paediatric DR in Australia and Saudi Arabia.

**Methods:**

A survey‐based study was devised and distributed to radiographers from Australia and Saudi Arabia. Questions focused on Australian and Saudi Arabian radiographers’ knowledge and attitude of paediatric DR examinations.

**Results:**

There were 376 participants who responded to the survey from both countries. A major finding showed that most participants lack knowledge in the area of paediatric DR examinations. Most participants from Australia had received no formal training in paediatric digital radiography (79%), whereas nearly half of the participants from Saudi Arabia received no training (45%). Approximately three out of four radiographers from both countries believed that when using DR they did not need to change the way they collimate the beam as DR images can be cropped using post‐processing methods.

**Conclusion:**

The finding of this study demonstrates that radiographers from both countries should improve their understanding and clinical use of DR in paediatric imaging. More education and training for both students and clinicians is needed to enhance radiographer performance in digital radiography and improve their clinical practices.

## Graphical abstract

The purpose of this work was to evaluate the knowledge, awareness and attitudes of radiographers with paediatric digital radiography in terms of image quality optimisation between radiographers in Australia and Saudi Arabia. This study demonstrates that radiographers from both countries should endeavour to improve their perceptions and understanding of paediatric digital radiography. More education and training is recommended to enhance radiographer performance in paediatric digital radiography.

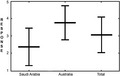

## Introduction

Digital radiography (DR), which includes computed radiography (CR), indirect digital radiography (IDR) and direct digital radiography (DDR), is the latest planar medical imaging technology that has transformed medical imaging practices. The change from screen‐film radiography (SFR) to DR has positively influenced clinical productivity, image quality optimisation and diagnostic interpretation.^1^ Introducing these newer imaging systems could potentially aid in reducing the radiation dose to patients.^2^ Despite this, patients may be exposed to higher radiation doses than are required for suitable image quality if radiographers’ practices are not adjusted and corrected.^3^, ^4^, ^5^

One of the main concerns in DR is exposure creep. Exposure creep is where radiographers, over a period of time, use higher exposure factors than are required for appropriate image quality.^6^ The wide dynamic range of DR systems has potential advantages in that over‐ or underexposures can be adjusted electronically using post‐processing functions of brightness/ contrast controls.^7^, ^8^ However, this wide dynamic range also encourages the use of higher radiation doses as noise is reduced with higher exposure factors, hence improving image quality. Without paying attention to the exposure factors required for each individual patient, radiographers can increase radiation dose to their patients. In DR, increasing radiation to the image plate will result in better image quality without necessarily improving diagnostic ability.^7^

The exposure factors and methods of optimisation used with digital radiographic images are different from the methods used with radiographic films.^9^ There are different guidelines in optimising image quality due to the range variety of DR technologies that have distinctive imaging methods.^3^, ^4^ Researchers have reported that due to the inadequate knowledge and lack of experience with DR, radiographers could potentially expose patients to unnecessary doses of radiation.^4^, ^10^ It is highly recommended that radiographers receive more education in order to benefit effectively from DR.^9^, ^11^, ^12^ Radiographers have to minimise the dose of radiation that patients are exposed to while still maintaining image quality. In order to produce the ideal image quality, radiographers are required to have proper exposure factors.^13^ There should be specific safety measurements while dealing with paediatric patients.^14^, ^15^ The following parameters that should be dealt with caution and extra care are the X‐ray tube potential (kVp), tube current (mA), exposure time (s), source to image distance (SID), focal spot size and many more. Radiographers have to have a good knowledge of all exposure factors and technical parameters in relation to image quality optimisation and dose minimisation in DR.^16^

The potential for increased radiation dose in paediatric DR was recognised early in the introduction of these imaging modalities.^17^, ^18^ Since these articles were published, little change to the practice of radiography in paediatric DR examinations has occurred.^19^, ^20^ Understanding of current issues of optimising image quality and reducing the radiation dose in paediatric radiography is essential to improve radiographers’ performance when imaging children. This is in agreement with the Imaging Gently Campaign.^21^ The main purpose of this study was to evaluate radiographers’ knowledge, awareness and attitude of image quality optimisation and radiation dose management in paediatric DR. The countries chosen for the survey were Saudi Arabia and Australia. These two countries were chosen as the researchers are based in these countries where participants could be sought to respond to the survey. Further, any outcomes from this work could potentially benefit paediatric patients in these countries.

## Materials and Methods

### Ethics and questionnaire design

A cross‐sectional study design was chosen for the evaluation of and comparison between the Australian and Saudi Arabian radiographers’ knowledge and attitude of paediatric DR. The study was approved by Institutional Review Board (IRB) of the Imam Abdulrahman Bin Faisal University (IRB‐2015‐04‐068). The requirement for ethics approval was waived in Australia by the University of Canberra Human Ethics Committee due to IRB approval of an international survey and as such covering Australia.

A pilot of the survey was undertaken in Saudi Arabia. Some minor changes to the questionnaire were made following the feedback from the pilot study. The finalised survey contained four sections. The first section’s questions focused on demographic information. The other three sections included questions that measured radiographers’ knowledge of and attitude to optimising radiation dose and image quality for DR. Questions were included to evaluate the knowledge and attitude of radiographers in paediatric X‐ray examinations. The survey principally used closed‐ended questions; however, some open‐ended questions were included to permit broader participants’ response.^22^

### Recruitment and data collection

In Australia, survey information and recruitment of participants was sent via the Australian Society of Medical Imaging and Radiation Therapy (ASMIRT) email system to all members. The survey was opened to participants in the first half of 2016 for online completion, and the data were collected electronically.

In Saudi Arabia, participants’ access to online surveys is problematic due to Internet not being broadly available or access‐restricted in the workplace. The approach used in Saudi Arabia was to contact the heads of radiological departments in hospitals, which undertake paediatric radiography. With the head of departments’ approval and already gained Institutional Review Board ethics, the management of four hospitals approved surveying radiographers in their hospital. Packages of hard copy surveys were sent to each hospital department head, which included an invitation to participate in the survey, a plain language statement and the questionnaire. The head of each department was requested to distribute the questionnaire copies to the radiographers in the department. After completing the questionnaire, participants were asked to give the completed questionnaire form to the researcher to ensure confidentiality.

The total number of radiographers who participated in the survey from Australia was 298 and in Saudi Arabia was 78.

### Data analysis

Saudi Arabian and Australian radiographers’ knowledge and attitudes about paediatric DR were analysed and compared by using t‐test, chi‐squared test in SPSS (version 18 – SPSS Inc., Chicago, USA). *P*‐value was calculated using t‐test: two samples assuming unequal variances in Microsoft Office Excel to judge if there were significant differences between radiographers’ responses. The differences were considered significant if the *P*‐value was less than 0.05.

## Results

The results presented are a subset of a broad‐based survey of radiographers’ knowledge of and awareness and attitudes in paediatric digital radiography. Three hundred and seventy‐six radiographers participated from different hospitals in Australia and Saudi Arabia. Table 1 provides the demographic characteristics of the participants.

**Table 1:** Demographic data of the participants (*n* = 376).

		Number of respondents (%)		
		Saudi Arabia	Australia	Total
Age	Under 25	4 (6.2%)	23 (7.7%)	27 (7.4%)
	25–35	34 (52.3%)	115 (38.6%)	149 (41%)
	36–45	8 (12.3%)	61 (20.5%)	69 (19%)
	46–55	8 (12.3%)	57 (19.1%)	65 (17.9%)
	Older than 55	11 (16.9%)	42 (14.1%)	53 (14.6%)
Highest academic qualification	Diploma	20 (25.6%)	54 (18.1%)	74 (19.7%)
	College diploma	28 (35.9%)		28 (7.4%)
	Bachelor	29 (37.2%)	182 (61.1%)	211 (56.1%)
	Master	1 (1.3%)	55 (18.5%)	56 (14.9%)
	PhD		3 (1%)	3 (0.8%)
	None of the above		4 (1.3%)	4 (1.1%)
Years of experience in computed radiography (CR).	< 5	6 (7.7%)	80 (26.8%)	86 (23.9%)
	5 to 10	16 (25.8%)	112 (37.6%)	128 (35.6%)
	10 to 15	21 (33.9%)	63 (21.1%)	84 (23.3%)
	> 15	14 (22.6%)	39 (13.1%)	53 (14.7%)
	None	5 (8.1%)	4 (1.3%)	9 (2.5%)
Years of experience in computed radiography, in direct digital radiography (DDR) or indirect digital radiography (IDR).	< 5	5 (8.1%)	154 (51.7%)	159 (44.2%)
	5 to 10	18 (29%)	85 (28.5%)	103 (28.6%)
	10 to 15	21 (33.9%)	21 (7%)	42 (11.7%)
	>15	11 (17.7%)	10 (3.4%)	21 (5.8%)
	None	7 (11.3%)	28 (9.4%)	35 (9.7%)
Formal training in digital radiography (DR).	Yes	48 (61.5%)	119 (40.3%)	167 (44.8%)
	No	30 (38.5%)	176 (59.7%)	206 (55.2%)
Formal training in radiation safety of digital radiography.	Yes	34 (44.7%)	85 (28.7%)	119 (32.0%)
	No	42 (55.3%)	211 (71.3%)	253 (68.0%)
Participant’s concerns about radiation dose	Yes	37 (48.7%)	96 (32.2%)	133 (35.6%)
	No	39 (51.3%)	202 (67.8%)	241 (64.4%)
Familiar with the ALARA (as low as reasonably achievable/acceptable) principle.	Yes	52 (71.2%)	289 (97%)	341 (91.9%)
	No	21 (28.8%)	9 (3%)	30 (8.1%)

**Figure 1:**
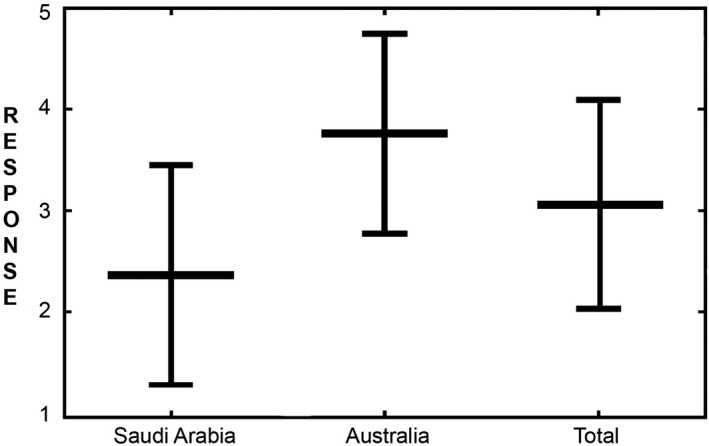
Participants’ responses to the question ‘Digital radiography has a potential problem of dose creep which increases the radiation dose over time’. Responses from 1 (strongly agree) to 5 (strongly disagree).

The participants were asked to respond to the statement ‘DR has problems of dose creep which increases the radiation dose over time’ where a response of 1 means strongly disagree, 2 means disagree, 3 means neutral, 4 means agree, and 5 means strongly agree. Figure 1 shows responses from Saudi Arabia and Australia and the combined responses. Figure 1 shows the mean values and ranges of the responses for visual information only.

The participants were asked to respond ‘Yes’ or ‘No’ to several statements describing their practice of radiography. These questions were devised to evaluate the participant’s knowledge and attitude of image quality optimisation and dose managements. The statements and responses can be seen in Table 2. The significance of the difference between participants’ responses from Saudi Arabia and Australia, shown as *P*‐values, is provided.

**Table 2:** Respondents’ responses to questions on their perception about image optimisation factors

		Saudi Arabia	Australia	Total	*P*‐values
Digital radiography changes the way beam collimation is needed as images can be cropped in digital radiography	Yes	43 (63.2%)	41 (15.6%)	84 (25.5%)	0.000
	No	25 (36.8%)	221 (84.4%)	246 (74.5%)	
I rely more on image cropping than collimation to avoid cutting region of interest and hence avoid exposure repetition	Yes	31 (45.6%)	31 (11.9%)	62 (18.8%)	0.000
	No	37 (54.4%)	230 (88.1%)	267 (80.9%)	
Collimating the X‐ray beam leads to unnecessary radiation dose burden to the patient	Yes	40 (60.6%)	51 (19.5%)	91 (27.8%)	0.000
	No	26 (39.4%)	210 (80.5%)	236 (72.2%)	
Have you received formal training in paediatric digital radiography?	Yes	18 (23%)	54 (20.8%)	72 (28.2%)	0.000
	No	35 (44.9%)	205 (78.8%)	240 (58.6%)	
	Not Sure	25 (32%)	1 (0.4%)	26 (13.1%)	
Grids are used when patient size is more than 8 to 10 cm in thickness	Yes	36 (46.2%)	56 (23%)	92 (28.6%)	0.000
	No	26 (33.3%)	147 (60.2%)	173 (53.7%)	
	Not Sure	16 (20.5%)	41 (16.8%)	57 (17.7%)	

To evaluate what radiographers consider is applicable in the management of radiation dose in their clinical practice, participants were asked to respond ‘Yes’ or ‘No’ to the statements provided in Table 3. Table 3 shows the responses from Saudi Arabian, Australian and combined responses. The significance of the difference between participants’ responses from Saudi Arabia and Australia, shown as *P*‐values, is provided.

**Table 3:** Respondents’ responses to questions on their perception on the importance of managing radiation dose

	Countries	Number of respondents (%)			
		Yes	No	Not sure	*P*‐values
Monitoring your repeat rate	Saudi Arabia	64 (82.1%)	2 (2.6%)	12 (15.4%)	0.001
	Australia	244 (92.8%)	15 (5.7%)	4 (1.5%)	
	Total	308 (90.3%)	14 (4.1%)	16 (4.7%)	
Using X‐ray beam collimation	Saudi Arabia	70 (89.7%)	0 (0.0%)	8 (10.3%)	0.003
	Australia	259 (98.1%)	4 (1.5)	1 (0.4%)	
	Total	326 (96.2%)	8 (2.4%)	9 (2.7%)	
Monitoring and considering the exposure index	Saudi Arabia	56 (73.1%)	9 (11.5%)	12 (15.4%)	0.000
	Australia	253 (95.8%)	9 (3.4%)	2 (0.8%)	
	Total	309 (90.6%)	21 (6.2%)	14 (4.1%)	
Adjusting exposure factors to avoid unnecessary radiation dose	Saudi Arabia	62 (79.5%)	6 (7.7%)	10 (12.8%)	0.000
	Australia	258 (97.7%)	5 (1.9%)	1 (0.4%)	
	Total	320 (93.6%)	16 (4.7%)	11 (3.2%)	
Using automated exposure factors and electronic collimation	Saudi Arabia	44 (56.4%)	18 (23.1%)	16 (20.5%)	0.000
	Australia	130 (50%)	120 (46.2%)	10 (3.8%)	
	Total	174 (51.5%)	34 (10.1%)	26 (7.7%)	
Using validated radiographic technique charts as a function of patient size for all performed examinations to avoid dose creep	Saudi Arabia	64 (82.1%)	4 (5.1%)	10 (12.8%)	0.002
	Australia	188 (71.5%)	61 (23.2%)	14 (5.3%)	
	Total	252 (73.9%)	14 (4.1%)	24 (7%)	
Routinely updating the exposure factors to obtain optimum image quality and avoid excessive radiation dose	Saudi Arabia	65 (83.3%)	5 (6.4%)	8 (10.3%)	0.057
	Australia	198 (75.3%)	47 (17.9%)	18 (6.8%)	
	Total	263 (77.1%)	13 (3.8%)	26 (7.6%)	
Using a higher kVp and lower mAs	Saudi Arabia	55 (70.5%)	5 (6.4%)	18 (23.1%)	0.002
	Australia	173 (65.8%)	62 (23.6%)	28 (10.6%)	
	Total	228 (66.9%)	23 (6.7%)	46 (13.5%)	

To evaluate radiographer’s understanding of paediatric radiography, participants were asked to respond with ‘True’ or ‘False’ to concepts in paediatric radiography. The conceptual statements and responses are provided in Table 4. The significance of the difference between participants’ responses from Saudi Arabia and Australia, shown as *P*‐values, is provided.

**Table 4:** Respondents’ responses to stated concepts in paediatric radiography

		Number of respondents (%)			
	Countries	True	False	Not sure	P‐values
Paediatric radiography has imaging challenges that differ from typical adult radiography.	Saudi Arabia	73 (93.6%)	0 (0.0%)	5 (6.4%)	0.031
	Australia	246 (98.4%)	3 (1.2%)	1 (0.4%)	
	Total	319 (97.3%)	3 (0.9%)	6 (1.8%)	
When imaging, paediatric patients are believed to be up to double times more sensitive to ionising radiation than adults	Saudi Arabia	42 (53.8%)	26 (33.3%)	16 (12.8%)	0.000
	Australia	95 (38.2%)	72 (28.9%)	82 (32.9%)	
	Total	137 (41.9%)	98 (30%)	92 (28.1%)	
Paediatric patients are ten times more sensitive to ionising radiation than adults	Saudi Arabia	30 (38.5%)	31 (39.7%)	17 (21.8%)	0.000
	Australia	87 (35.1%)	51 (20.6%)	110 (44.4%)	
	Total	117 (35.9%)	82 (25.2%)	127 (39%)	
The risk of cancer mortality attributable to a single, acute radiation exposure for patients under 15 years of age is more than twice the average risk for patients in other age cohorts	Saudi Arabia	43 (55.1%)	17 (21.8%)	18 (23.1%)	0.002
	Australia	126 (50.4%)	26 (10.4%)	98 (39.2%)	
	Total	169 (51.5%)	43 (13.1%)	116 (35.4%)	
The same radiographic techniques (kVp, mAs, SID, collimation, image processing algorithm, etc.) used for adults can be applied to paediatrics	Saudi Arabia	31 (39.7%)	37 (47.4%)	10 (12.8%)	0.000
	Australia	46 (18.4%)	201 (80.4%)	3 (1.2%)	
	Total	77 (23.5%)	238 (72.6%)	13 (4%)	

The final question of the survey was an open‐ended question. The question asked respondents to provide causes of excessive radiation exposure when performing paediatric DR imaging. The only responses received were from Australian respondents (Table 5).

**Table 5:** Respondents’ responses to questions about which factors cause excess radiation exposure when performing paediatric examinations?

	Number of responses	
	CR	DR
Uncooperative patient/motion	76/318	51/264
Unnecessary X‐ray examination	20/318	17/264
Inappropriate exposure factors	78/318	53/264
Lack of training/knowledge	55/318	68/264
Lacking or improper immobilisation	18/318	9/264
Poor collimation	32/318	20/264
Malfunction of equipment	2/318	2/264
Unnecessary repetition	4/318	28/264
Others (patient size, using grids, no evaluation of exposure time to time, improper use of post‐processing, complexity of equipment or malfunctions, misuse of lead shielding, automatic exposures, laziness of radiographers	33/318	16/264

## Discussion

This work is part of a broad‐based survey of radiographers’ knowledge of and awareness and attitudes in paediatric digital radiography. The focus of this work was to gain an understanding of Saudi Arabian and Australia radiographers’ knowledge, awareness and attitudes to paediatric digital radiography. To achieve this goal, the researcher also needed an understanding of the radiographers’ knowledge and understanding of the imaging modalities of CR, DDR and IDR; hence, some questions were asked around these specific areas.

Specific findings that are relevant to this work are in the following subsections.

### Participants’ demographic characteristics

The majority of the Australia participants, 61%, and a high proportion of participants from Saudi Arabia, 37%, had a bachelor’s degree or higher qualifications as highest level of study. Approximately 20% of participants from both countries had no experience at all in CR, DDR and IDR. This may reflect two issues. First, the biggest group of participants are in the two younger age groups, and second, DDR and IDR are still being introduced as the main imaging modality. It is noteworthy that more than half of the total number of participants (59%) received no formal training in paediatric DR.

### Image quality improvement and dose management in paediatric imaging

To assess participants’ knowledge and understanding of image quality and optimisation, the statement ‘DR has problems of dose creep which increases the radiation dose over time’ was put to respondents. The respondents generally agreed with the statement. However, Saudi Arabian respondents most likely disagreed and Australian respondents most likely agreed (see Figure 1).

Participants were asked if they monitor and consider exposure indices in paediatric DR. The majority of participants (90.6%) affirmed that they use exposure indices to monitor the dose they deliver to their paediatric patients.

To assess participants’ knowledge and understanding of quality improvement processes, questions were asked around repeat rates and validating and updating radiographic exposure factors and technique charts. There was a strong positive affirmation that quality improvement processes were undertaken in paediatric imaging.

Mc Fadden et al^16^ concluded in their overview of radiographic practice in Europe that radiographers need to have good knowledge of technical factors relating to patient dose and image quality. It is evident from this survey that more focus is needed to increase this understanding in both Saudi Arabia and Australia.

### Knowledge and attitude of image quality optimisation and dose management

The majority of the participants (74.5%) indicated that when using DR they did not need to change the way they collimate the beam as in DR images can be cropped using post‐processing methods. About 84.4% of participants from Australia and 63.2% of participants from Saudi Arabia showed that when using DR, they did not change the way they use beam collimation (see Table 2). Nearly, all participants from Saudi Arabia and Australia (95.8% and 98.9%, respectively) reported that the collimating of the X‐ray beam is still important in DR. The results also showed that the majority of the participants (80.9%) did not rely on image cropping to avoid cutting off anatomical regions.

The responses from the two questions, ‘Digital radiography changes the way beam collimation is needed as images can be cropped in digital radiography’ and ‘I rely more on image cropping than collimation to avoid cutting region of interest and hence avoid exposure repetition,’ are at odds to each other. The two questions are essentially the same, yet responses are not consistent. This may imply that radiographers generally do not fully understand the difference between physical collimation and digital cropping.

Collimation is an essential technique in conventional radiography and in DR, to limit the amount of tissue irradiated and to maintain lower radiation dose to patients. Collimation technique also reduces scatter radiation, and consequently, the image contrast is enhanced; thus, the image quality is improved.^23^, ^24^ Zabihzadeh and Karami^25^ reported that gonadal dose can be increased by improper collimation. In other studies, researchers suggest that the highest unnecessary dose to patients is due to inadequate collimation.^11^, ^26^

Most Saudi Arabian radiographers and a considerable percentage of Australian radiographers believe that the introduction of DR changed the importance of beam collimation. For example, nearly half of Saudi participants relied on image cropping more than collimation. In comparison, Australian participants have a better understanding of the importance of beam collimation than the Saudi Arabian participants. This may be explained by that the Australian participants have higher academic qualification levels than Saudi Arabian participants do, even though the results showed that Saudi Arabian participants have more experience with DR.

The results of the current study are comparable with several studies conducted previously. The survey study conducted by Morrison et al^11^ found that half of 493 participant radiographers used electronic image cropping more than 75% of the time during paediatric radiography.

Other studies have reported that collimation in DR tends to be larger than needed.^27^, ^28^

It is suggested that electronic cropping of digital images may be the reason why the radiographers are complacent towards proper collimation as they may use large collimation then they can mask unwanted borders of image.^1^, ^27^

Exposure field recognition errors are most likely to arise when the X‐ray exposure field is wrongly collimated and positioned.^29^ Pre‐processing histogram analysis can result in dark or light images and can lead to the addition of noise in the image. Improper collimation, incorrect field size and positioning may cause recognition errors.^29^ These errors lead to incorrect histogram analysis as the signal outside the exposure field is involved in histogram calculations, and consequently, artefacts occur. The images obtained with these errors can result in dark, light or noisy images. In addition, the sensitivity of image receptors increases the risk of scatter radiation impacting on image quality.^28^ Incorrect collimation can lead to increased scatter and a reduction of image quality. Further, incorrect collimation of exposure field influences the radiation dose delivered to the patient.^8^, ^23^, ^30^ For example when the collimation is unnecessarily opened, the dose to the patient consequently increases.^20^, ^31^, ^32^

In addition, most of the participants from Australia and Saudi Arabia confirmed that they did not receive formal training in radiation protection. Such training courses are important to increase the awareness and knowledge of radiographers about the role of beam collimation in radiation dose reduction. Image cropping cannot replace beam collimation. Radiation dose can be minimised to the patient by using collimation and other techniques.

### Knowledge and attitude in paediatric imaging

A large proportion of the participants from Saudi Arabia (45%) had not received any formal training in paediatric imaging in DR. In addition, 32% of them were not sure if they received training or not. A larger proportion, 79%, of the participants from Australia received no formal training in paediatric DR (see Table 2).

Participants from Saudi Arabia and Australia had a similar percentage of respondents who agreed with the statement ‘Imaging paediatric patients are believed to be up to ten times more sensitive to ionising radiation than adults’ (38% and 35%, respectively). However, the majority of participants from both countries were unsure or disagreed with the statement (see Table 4).

A large percentage (46%), see Table 2, of participants from Saudi Arabia agreed with the statement ‘Grids are used for patients with size more than 8 or 10 cm thickness’. However, 60% of participants from Australia disagreed with this statement. More than half of the participants from Saudi Arabia agreed that using grids is only required for abdomen, spine, pelvis, skull and cross‐table lateral radiographs. However, only 45% of participants from Australia agreed with this statement. Most participants from Saudi Arabia and Australia agreed that grids are not necessary for infants or small children (64% and 85%, respectively). Nearly half of participants from Saudi Arabia and the majority of the participants from Australia (49% and 54%, respectively) disagreed with using grids for all examinations.

The outcomes of this aspect of the survey show a variety of different results in the knowledge, attitudes and practices between radiographers. A further example of the variation in knowledge and attitudes is that there was a high level of agreement that ‘paediatric radiography has imaging challenges that differ from typical adult radiography’ yet a converse statement on a similar topic of ‘the same radiographic techniques (kVp, mAs, SID, collimation, image processing algorithm, etc.) used for adults can be applied to paediatrics’ shows a lower level of disagreement with this statement. Table 4 shows the details of these survey questions. A recent study conducted by Mc Fadden et al^16^ suggested that there is a wide variation in radiographer education and training across European countries. The results of this survey are similar to their findings.

### Responses to open question of the survey form

Requesting unnecessary X‐ray paediatric examinations was determined by some participants as a factor in increasing the radiation dose in CR and DR. Examples of such examinations include plain abdominal radiographs to diagnose idiopathic constipation; imaging in unnecessary surgery medial pinning of supracondylar fractures; imaging for acute pneumonia; and the daily chest radiographs in ICU.

The data collected from the participants provided some explanation as to why the radiographers use inappropriate exposure factors. These include over‐reliance on pre‐programmed exposure factors and pressure not to repeat the exposure. Some participants stated that radiographic equipment is frequently installed without further training, and radiographers just adopt the same exposure techniques as they were using before.

Other factors that can lead to higher radiation dose were provided by participants and included such things as using grids for smaller children; complexity and malfunctions of equipment; laziness of some practitioners; and poor centring. Using automatic exposures with the chamber not in the correct area and the limited understanding of how to use manual exposures were also provided as causes of repeated exposures and hence higher dose to patients.

Participants reported that the major factors that cause the excess radiation dose to patients in CR are inappropriate setting of exposure factors, motion and lack of training and knowledge (25%, 24% and 17%, respectively). For DR, the results showed similar factors but with different influencing levels. The highest influencing level factor was lack of training and knowledge, then inappropriate exposure factors and then the patient motion (26%, 20% and 19%, respectively). Some participants emphasised the role of lack of knowledge in increasing the dose to patient. For example, an expert radiographer in clinical practice and teaching stated that:

I believe that there is an ever INCREASING lack of knowledge of the function and use of specific exposure factors. i.e. kVp, mA & time & the effect that they have on image quality and radiation dose. CR/DR masks too many mistakes and some radiographers have little to no concept on correct exposure factor usage and/ or how to correct/ adjust for individual patient size and age.

### Limitations

The limitations of this work are several. The survey was only undertaken in two countries. As such, the finding and recommendation can only be applied to these two countries. The number of Saudi participants, who were from only four hospitals, is a limitation as paediatric studies are performed in a wider number of hospitals. It is therefore recommended to include all hospitals in Saudi Arabia in future surveys so to include as many as possible Saudi radiographers. The pilot study of this work was only undertaken in Saudi Arabia. Undertaking a pilot study in Australia may have assisted in clarification of some of the questions.

## Conclusion

This study generally reveals the need of radiographers to undertake further training and gain additional knowledge to enhance their performance in paediatric DR examinations in both countries where the survey was undertaken. This survey has revealed that with the introduction of DR systems, the motivation towards adequate collimation seems to be reduced. Radiographers’ understanding of exposure factors and how these influence image quality and dose in paediatric DR also should be improved. A focus of this understanding, for example, should be to emphasise the importance of beam collim ation to optimise image quality while maintaining lower radiation dose to patient.

Education in these areas should be implemented, revised or updated in university programmes and in the workplace. Universities and workplace programmes should emphasise the knowledge of best practice of digital radiography and clarify the common malpractices of radiographers in paediatric DR. It is also important to continuously follow the improvement and development of radiographers’ quality of radiographic examinations by directly monitoring performance and images by regular quality assurance checking of images and data, such as exposure indices, from the picture archival and communication systems (PACS).

It is recommended that similar studies to this should be repeated at regular intervals to examine the perception and knowledge of radiographers in paediatric radiography.

## References

[1] Herrmann TL , Fauber TL , Gill J , et al. Best practices in digital radiography. Radiol Technol 2012; 84: 83–9. 22988267

[2] Weatherburn GC , Ridout D , Strickland NH , et al. A comparison of conventional film, CR hard copy and PACS soft copy images of the chest: analyses of ROC curves and inter‐observer agreement. Eur J Radiol 2003; 47: 206–14. 12927664 10.1016/s0720-048x(02)00214-0

[3] Körner M , Weber CH , Wirth S , Pfeifer K‐J , Reiser MF , Treitl M . Advances in digital radiography: physical principles and system overview. Radiographics 2007; 27: 675–86. 17495286 10.1148/rg.273065075

[4] Williams MB , Krupinski EA , Strauss KJ , et al. Digital radiography image quality: image acquisition. J Am Coll Radiol 2007; 4: 371–88. 17544139 10.1016/j.jacr.2007.02.002

[5] Kei W , Hogg P , Norton S . Effects of kilovoltage, milliampere seconds, and focal spot size on image quality. Radiol Technol 2014; 85: 479–85. 24806050

[6] Gibson DJ , Davidson RA . Exposure creep in computed radiography: a longitudinal study. Acad Radiol 2012; 19: 458–62. 22225727 10.1016/j.acra.2011.12.003

[7] Seeram E , Davidson R , Bushong S , Swan H . Optimizing the exposure indicator as a dose management strategy in computed radiography. Radiol Technol 2016; 87: 380–91. 26952062

[8] Fauber TL , Cohen T , Dempsey M . High kilovoltage digital exposure techniques and patient dosimetry. Radiol Technol 2011; 82: 501–10. 21771934

[9] Alsleem H , Davidson R . Quality parameters and assessment methods of digital radiography images. Radiographers 2012; 59: 46–55.

[10] Schaefer‐Prokop C , Neitzel U , Venema HW , Uffmann M , Prokop M . Digital chest radiography: an update on modern technology, dose containment and control of image quality. Eur Radiol 2008; 18: 1818–30. 18431577 10.1007/s00330-008-0948-3 PMC2516181

[11] Morrison G , John SD , Goske MJ , et al. Pediatric digital radiography education for radiologic technologists: current state. Pediatr Radiol 2011; 41: 602–10. 21491200 10.1007/s00247-010-1904-3

[12] Alsleem H , U P, Mong KS, Davidson R . Effects of radiographic techniques on the low‐contrast detail detectability performance of digital radiography systems. Radiol Technol 2014; 85: 614–22. 25002641

[13] International Society of Radiographers and Radiological Technologists . Guidelines for the Education of Entry‐level Professional Practice in Medical Radiation Sciences. International Society of Radiographers and Radiological Technologists, London, 2004.

[14] Australian Society of Medical Imaging and Radiation Therapy . Code of Ethics. Australian Society of Medical Imaging and Radiation Therapy, Melbourne, Australia, 2017.

[15] Scott AM . Current issues in radiation dose monitoring and reporting. Radiol Technol 2014; 85: 501–16. 24806053

[16] Mc Fadden S , Roding T , de Vries G , Benwell M , Bijwaard H , Scheurleer J . Digital imaging and radiographic practise in diagnostic radiography: An overview of current knowledge and practice in Europe. Radiography 2018; 24: 137–41. 29605110 10.1016/j.radi.2017.11.004

[17] Willis CE , Slovis TL . The ALARA Concept in Pediatric CR and DR: Dose Reduction in Pediatric Radiographic Exams — A White Paper Conference. Am J Roentgenol. 2005; 184(2): 373–4. 15671348 10.2214/ajr.184.2.01840373

[18] Willis CE . Strategies for dose reduction in ordinary radiographic examinations using CR and DR. Pediatr Radiol 2004; 34: S196–S200. 15558261 10.1007/s00247-004-1269-6

[19] Don S . Pediatric digital radiography summit overview: state of confusion. Pediatr Radiol 2011; 41: 567–72. 10.1007/s00247-010-1905-2 21491196

[20] Karami V , Zabihzadeh M , Gilavand A , Shams N . Survey of the use of x‐ray beam collimator and shielding tools during infant chest radiography. Int J Pediatr 2016; 4: 1637–42.

[21] The Image Gently Alliance . Image Gently Campaign. 2018. [cited 2018 May 30] Available from: https://www.imagegently.org/.

[22] Zuley ML . The basics and implementation of digital mammography. Radiol Clin North Am 2010; 48: 893–901. 20868892 10.1016/j.rcl.2010.06.003

[23] Bushong S . Radiologic Science for Technologists: Physics, Biology and Protection , 11th: edn. Elsevier, St Louis, MO, 2016.

[24] Khong PL , Ringertz H , Donoghue V , et al. ICRP publication 121: radiological protection in paediatric diagnostic and interventional radiology. Ann ICRP 2013; 42: 1–63. 10.1016/j.icrp.2012.10.001 23218172

[25] Zabihzadeh M , Karami V . Letter to Editor: Poor collimation in digital radiology: A growing concern. Internet J Med Update 2016; 11: 29–30.

[26] Okeji M , Anakwue A , Agwuna K . Radiation exposure from diagnostic radiography: an assessment of X‐ray beam collimation practice in some Nigerian Hospitals . Int J Med Update 2010; 5: 31–3.

[27] Zetterberg LG , Espeland A . Lumbar spine radiography–poor collimation practices after implementation of digital technology. Br J Radiol 2011; 84: 566–9. 21606070 10.1259/bjr/74571469 PMC3473630

[28] Debess J, Johnsen K, Vejle Sørensen K, Thomsen H, Aalborg Øst DKV Digital chest radiography: collimation and dose reduction. European Congress of Radiology 2015; 2015; Vienna: European Society of Radiology.

[29] Uffmann M , Schaefer‐Prokop C . Digital radiography: The balance between image quality and required radiation dose. Eur J Radiol 2009; 72: 202–8. 19628349 10.1016/j.ejrad.2009.05.060

[30] Shetty CM , Barthur A , Kambadakone A , Narayanan N , Kv R . Computed Radiography Image Artifacts Revisited. Am J Roentgenol 2011; 196(1): W37–W47. 21178029 10.2214/AJR.10.5563

[31] Robinson JB , Ali RM , Tootell AK , Hogg P . Does collimation affect patient dose in antero‐posterior thoraco‐lumbar spine? Radiography 2017; 23: 211–5. 28687288 10.1016/j.radi.2017.03.012

[32] Walz‐Flannigan AI , Brossoit KJ , Magnuson DJ , Schueler BA . Pictorial review of digital radiography artifacts. Radiographics 2018; 38: 833–46. 29676963 10.1148/rg.2018170038

